# Management of non-traumatic abdominal pain in the emergency department: a multicentre, stepped-wedge, cluster-randomised trial

**DOI:** 10.1016/j.lanepe.2025.101362

**Published:** 2025-06-26

**Authors:** Anna Slagman, Liane Schenk, Dörte Huscher, Lisa Arnold, Harald Dormann, Johannes Drepper, Larissa Eienbröker, Antje Fischer-Rosinský, Johann Frick, Lukas Helbig, Dirk Horenkamp-Sonntag, Freddy Irorutola, Tim Klinge, Thomas Reinhold, Peter Schily, Britta Stier, Katharina Verleger, Andreas Wagenknecht, Yves Noel Wu, Martin Möckel

**Affiliations:** a Emergency and Acute Medicine, Charité – Universitätsmedizin Berlin, corporate member of Freie Universität Berlin and Humboldt-Universität zu Berlin, Charitéplatz 1, Berlin 10117, Germany; b Institute of Medical Sociology and Rehabilitation Science, Charité – Universitätsmedizin Berlin, corporate member of Freie Universität Berlin and Humboldt-Universität zu Berlin, Charitéplatz 1, Berlin 10117, Germany; c Institute of Biometry and Clinical Epidemiology, and Berlin Institute of Health, Charité – Universitätsmedizin Berlin, corporate member of Freie Universität Berlin and Humboldt-Universität zu Berlin, Charitéplatz 1, Berlin 10117, Germany; d Institute of Social Medicine, Epidemiology and Health Economics, Charité – Universitätsmedizin Berlin, corporate member of Freie Universität Berlin and Humboldt-Universität zu Berlin, Charitéplatz 1, Berlin 10117, Germany; e Emergency and Acute Medicine, Klinikum Fürth, Jakob-Henle-Straße 1, Fürth 90766, Germany; f TMF - Technology, Methods, and Infrastructure for Networked Medical Research, Charlottenstraße 42, Berlin 10117, Germany; g Techniker Krankenkasse, Bramfelder Straße 140, Hamburg 22305, Germany

**Keywords:** Abdominal pain, Emergency care, Emergency department, Acute care, Standardised treatment, Stepped wedge design

## Abstract

**Background:**

Even though abdominal pain is one of the most frequent chief complaints in emergency medicine, standardized care pathways are still lacking. In this study, a standardized, digitally-supported care pathway for non-traumatic abdominal pain in the emergency department was investigated with regard to emergency department length of treatment, pain score at discharge, and patient satisfaction.

**Methods:**

In a prospective mixed-methods, multicentre, cluster-randomised, controlled stepped wedge trial, adult patients with non-traumatic abdominal pain were enrolled in ten emergency departments across Germany. The new care pathway was implemented at randomly assigned time points (every four months) within a 24-month recruitment period and consisted of a structured care pathway for the management of abdominal pain patients in the emergency department. During the control period, the standard treatment for abdominal pain in the emergency department was administered. The planned sample size was 2000. Primary outcomes were: emergency department length of treatment, pain score (numeric rating scale 0–10), and patient satisfaction score at the end of emergency department treatment. Exploratory safety outcomes were serious adverse events within 30 days. Trial registration: DRKS00021052.

**Findings:**

Of 2119 patients, 1017 were enrolled in the control group, and 1102 in the intervention group. Crude mean emergency department length of treatment was 5.2 h (±3.0) in the control group, and 4.3 h (±2.2) in the intervention group while the adjusted mean difference was −0.31 h (95% confidence interval (CI) −0.70 to 0.07). Mean pain score was 4.3 (±2.5) in the control group, and 3.6 (±2.4) in the intervention group, resulting in an adjusted mean difference of −0.69 (95% CI −1.04 to −0.34). The adjusted mean difference of patient’s satisfaction score was 1.54 (95% CI 0.96 to 2.12); mean control group: 26.7 (±4.0); mean intervention group: 27.9 (±3.8)). Serious adverse events were comparable between both groups while 30-day mortality was 2.3% (n = 23) in the control group, and 0.8% (n = 9) in the intervention group (mean difference: −1.4% (95% CI −2.5 to −0.4)).

**Interpretation:**

The APU process is safe and did not increase emergency department length of treatment, while patient-reported outcomes and safety were improved accompanied by an increased use of diagnostic procedures.

**Funding:**

The study was funded by the German Innovations Funds.

**Research in context**

**Evidence before this study**

The authors considered all evidence related to abdominal pain patients in the emergency department. A scoping review was conducted in PubMed using the search terms abdominal pain and emergency. All studies referring to an emergency department population in English and German language were included but studies that examined trauma-related abdominal pain were excluded. No meta-analysis and risk of bias assessment was performed. The evidence was mainly descriptive and included analyses of emergency department presentations of abdominal pain patients and, in some cases, follow-up. Some analyses provided more insight into subgroups based on demographic characteristics. This search did not identify any standardised or interventional care pathways that were used or investigated.

**Added value of this study**

This is the first study to evaluate a new structured process for managing patients with non-traumatic abdominal pain in the emergency department. This study showed that the new treatment process increased patient satisfaction and reduced the pain score at the end of emergency department treatment, while the length of emergency department treatment did not increase compared to the control group. The results also suggest positive effects on patient safety and quality of care.

**Implications of all the available evidence**

This new structured abdominal pain treatment process in the emergency department is beneficial for patient self-reported outcomes, safety and quality of care and is recommended for widespread implementation. Future research should examine the impact of this process on populations underrepresented in this study (i.e., critically ill patients, ethnic minorities).

## Introduction

With a proportion of 5–20%, non-traumatic abdominal pain is one of the most common chief complaints in Emergency Departments.^1^, ^2^, ^3^, ^4^ Due to the heterogeneity and complexity of underlying diseases, non-traumatic abdominal pain is also one of the most challenging clinical symptoms in emergency medicine, and with 3–5%, in-hospital mortality is relatively high.^1^^,^^5^^,^^6^ Since some of the most common causes of death in patients with non-traumatic abdominal pain are time sensitive (e.g., sepsis, ileus, mesenteric infarction), these patients need to be managed in a fast and targeted way to improve clinical outcomes.^7^^,^^8^ There are no European guidelines for the management of patients with abdominal pain in general or atraumatic abdominal pain in particular in Europe. There may be local or regional care pathways for abdominal pain, but most care pathways are based on a suspected or confirmed diagnosis rather than a symptom and even though the use of diagnostic imaging increased substantially within the last decades, a high proportion of patients leaves the emergency department with a diagnosis of non-specific abdominal pain.^2^, ^3^, ^4^ A standardised and digitally supported diagnostic work-up could assure quality of care and reduce the time to final diagnosis and disposition in the emergency department in analogy to clinical decision support in cardiovascular medicine.^9^ The ‘Abdominal Pain Unit’ (APU) treatment process was developed to provide emergency department staff with a step-by-step standardised care pathway for non-traumatic abdominal pain patients from triage to disposition from the emergency department.^10^ The aim of the current study was to investigate the effect of the standardised treatment process for non-traumatic abdominal pain on emergency department length of treatment and patient reported outcomes: acute pain score and patient satisfaction with emergency department treatment at treatment completion in the emergency department.

## Methods

### Study design

A prospective mixed-methods, multicentre, cluster-randomised, controlled stepped wedge trial was conducted in ten study sites to compare routine clinical treatment of non-traumatic abdominal pain with the new digitally supported APU treatment process.^11^, ^12^, ^13^ Since the APU-process requires intense training of emergency department staff, a parallel-group design with individual patient-level randomisation was disregarded after thoughtful consideration to avoid a spillover effect of the intervention in the control group. All ten study centres started with recruitment in the control phase (routine clinical treatment), and sequentially implemented the digitally-supported APU process in randomly assigned clusters of two centres in four months intervals.^14^

### Study population

Adult patients with non-traumatic abdominal pain in the emergency department who were able to provide written informed consent for study participation were enrolled. For patients with a legal representative for medical decisions, written informed consent was obtained by both, the patients themselves, and the legal representative. Patients further needed to have statutory health insurance. Patients with trauma or with high suspicion of sepsis (quick Sequential Organ Failure Assessment^15^: qSOFA score ≥ 2) or shock (shock index ≥ 1) were excluded from study participation.

### Trial procedures

Recruitment took place after registration by administrative emergency department staff and initial standardised triage by nursing staff in the emergency department. Patients were screened by study nurses before first contact with the treating physician, and invited to participate by emergency department physicians if eligible for the study. Data were assessed at baseline (t0: index ED and hospital stay), and 30 days after initial emergency department admission (t1). In the control phase, patients were treated according to standard clinical practice at the respective emergency department. As per the randomly assigned clusters, the emergency departments switched from control to intervention phase. During the two-week implementation period, no patients were recruited, and the emergency department staff was trained in the digitally-supported APU process. The APU-process is a published clinical treatment process which guides clinical staff from initial patient evaluation through the entire diagnostic and clinical work-up until final diagnosis and specific treatment plan including the final patient disposition.^10^^,^^14^ There are underlying standard operating procedures for every step. The initial evaluation involves clinical status by vital parameters and Glasgow-Coma-Scale for qSOFA and shock-index. In case of suspected sepsis or shock, patients are referred to intensive care treatment and leave the APU-process. The first step in diagnostic work-up for all other patients is three-folded: (1) assessment of medical history, (2) clinical examination and (3) laboratory parameter evaluation. Furthermore, a pain assessment and respective pain management is recommended. If the diagnostic findings of the first step are inconclusive and do not allow for a working diagnosis and specific treatment measures, a sonography will be performed in the second step of the APU process. In the case of specific findings and diagnosis, patients can then be subjected to specific further care. Otherwise in a third step further diagnostic procedures are considered (e.g., additional imaging, multi-disciplinary consultation, observation). The patients leave the APU-process whenever the diagnostic findings allow for a specific diagnosis, treatment and disposition decision. The clinical status of the patients is monitored during the process and for those with worsening clinical condition appropriate care is administered.

A step by step flow diagram can be found in the Supplementary Appendix in Supplementary Fig. S1. The digital support was provided by an app that guided the treating physicians step by step through the APU process. The app could be used on handheld devices (e.g., mobile phone, tablet) or browser-based (developed by Realcore Group GmbH).

All enrolled patients were asked to fill out a written or tablet-based questionnaire (t0) within 72 h after treatment completion in the emergency department to assess patient reported outcomes. The survey could also be assisted by study personnel. Further secondary endpoints were assessed in a 30-day follow-up (t1). All trial procedures were tested and improved during a mono-centre pilot phase.

### Endpoints

The primary endpoints were emergency department length of treatment, and two patient-reported endpoints: acute pain score, and patient satisfaction with emergency department treatment. Emergency department length of treatment was defined as the time between first patient contact (first time stamp in the emergency department documentation system, e.g., triage or administrative admission), and treatment completion (time until final disposition decision by the attending physician). The patient-reported endpoints were assessed after treatment completion (≤72 h). Acute pain was assessed on a numeric rating scale 0–10,^16^ and patient satisfaction by eight questions with a rating of satisfaction on a four-point Likert scale regarding different dimensions of treatment satisfaction, which are combined into a sum score with a range between 8 and 32 points (ZUF-8^17^^,^^18^). The primary hypothesis of this study was that at least one endpoint would improve without deterioration of the other endpoints. Safety endpoints were serious adverse events as defined by the International Conference on Harmonisation Guideline for Good Clinical Practice (ICH-GCP), and were assessed during the initial hospital stay and within the 30-day follow-up period^19^: death, life-threatening events, unplanned or prolonged hospitalisation, disability or permanent damage, congenital anomaly, or birth defect. Exploratory endpoints included parameters concerning quality of care (quality of life,^20^ and patient’s general satisfaction at t0 and t1), process quality (process times, frequency of specific diagnostics, specific diagnoses), and subsequent utilisation of health care services (emergency department re-admission, re-admission to hospital, and utilisation of ambulatory health care services).

### Data assessment

Patients’ clinical characteristics, clinical course (process endpoints), and safety endpoints were assessed and documented in electronic case report forms (secuTrial, version 6.2.1.1, interActive Systems GmbH) for the entire index emergency department, and, if applicable, hospital stay. Patients were further re-contacted after 30 days (t1) via phone and online survey for additional assessment of clinical safety outcomes, further clinical course, and patient reported endpoints (e.g., quality of life: EUROHIS-QOL; range 1–5^20^^,^^21^).

### Statistical analysis

The intended sample size consisted of 2000 patients in total. Since the assessment of the primary endpoints was conducted at the end of emergency department treatment, a considerable dropout rate of up to 15% (n = 300) was expected. For the primary endpoints, the following assumptions were made: (1) Length of treatment in the emergency room: assumed reduction of 1 h from a mean of approx. 4.6 to 3.6 h (standard deviation 2.93). (2) Pain intensity on leaving the emergency department (numeric rating scale: 0–10): The estimated distribution based on emergency department data with 5239 patients corresponds to 0 (5%), 1 (5%), 2 (10%), 3 (20%), 4 (20%), 5 (20%), 6 (10%), 7–10 (10%, no more precise separation useful here due to low proportions). An increase in the skewness of the distribution in favor of better scores by 5% in categories 0 to 3, category 4 unchanged, corresponding decrease by 5% in categories 5 and 6, and by 10% in categories 7–10 was assumed. (3) Patient satisfaction (ZUF-8): improvement of 2 points from a mean of 26.9 to 28.9 points (standard deviation 4.01). A power of 1) 96%, 2) 99% and 3) 99% could be achieved for the assumed effect sizes with the achievable number of cases corrected for the SWD of n = 313 per treatment arm. The power estimation was based on the following assumptions: The intracluster correlation coefficients in human studies are usually between 0.01 and 0.02. By choosing the conservative variant of an intracluster correlation coefficients of 0.02, which leads to the highest SWD correction factor on this interval, we obtained a correction factor of 2.71. The application of this correction factor resulted in an effective total number of cases of n = 627, i.e., n = 313 per treatment arm, corresponding to an RCT design. For the power estimates, the significance level for 3 parallel Bonferroni tests was adjusted to α∗ = 0.05/3 = 0.0167. The primary hypothesis of this study was that at least one endpoint would improve without deterioration of the other endpoints. The power calculation included all three primary endpoints and was corrected for the stepped wedge-design with ten centres (nQuery 7.0, Statsols^22^).

For continuous variables, depending on an approximate normal distribution mean and standard deviation or median and interquartile range are reported; for categorical variables, counts and percentages, respectively. Decision for using non-parametric descriptive measures and tests was either driven by common sense, i.e., for duration of hospitalization or certain laboratory parameters, or graphically decided by evaluating boxplots. In the sample size calculation, we assumed random cluster effects, fixed time effects, but no interactions between cluster and time, thereby assuming that the variance of the cluster mean over time depends on changing participants, but that there is no time-dependent cluster variance. With further consideration of possibly non-normally distributed endpoints, including binary and ordinal data, or varying cluster sizes, the use of generalised linear mixed models was planned for the evaluation of the primary endpoints. In addition to the adjustment for centre effects, adjusted sensitivity analyses were pre-planned for unevenly distributed confounders that may occur despite randomization. Accordingly, generalised linear mixed models were calculated with fixed time effects for the step, and random centre effects (model I); since age turned out to be noticeably higher in the control group, sensitivity analyses were run also including age as additional fixed effect (model II). The health economic analysis is partly based on a certain sub-population of patients insured with a specific health insurance company (Techniker Krankenkasse) and is published separately.

### Ethical considerations

The study was funded by the German Innovations Funds (01NVF19025). The study protocol was reviewed and approved by the responsible institutional review boards and data protection commissioners of the participating study centres. The primary ethics approval was obtained at Charité–Universitätsmedizin Berlin (EA2/2019/20). The data protection concept was discussed and finally approved by the TMF's data protection working group. The trial was registered in the German Clinical Trials Registry (DRKS00021052) before initiation of enrollment and the study protocol was published.^14^ All serious adverse events were directly reported to the principal investigator and assessed for relation to the study procedures. The study was further monitored by an independent international advisory board. This manuscript follows the CONSORT-reporting guidelines for randomised controlled trials. A CONSORT-checklist is provided in the Supplementary Appendix.

### Role of the funding source

The study was funded by the German Innovations Funds (01NVF19025). The funding body conducted an independent peer review of the study protocol as part of the funding decision process but was not further involved in the design or conduct of the study.

## Results

### Study population

**Fig. 1:**
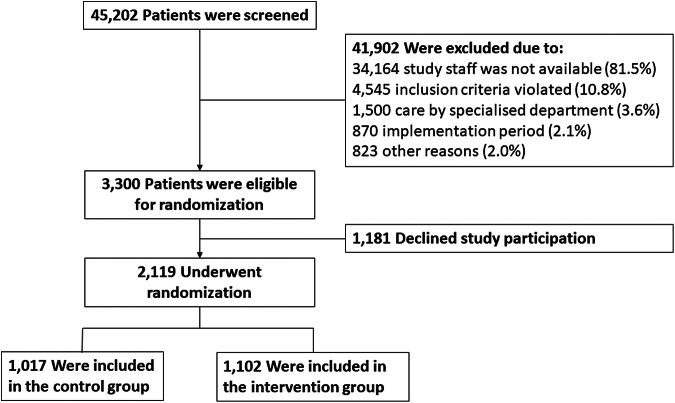
Enrollment and randomization of patients. A total of 45,202 patients were screened for eligibility and 41,902 were excluded. The primary reason for exclusion was that study staff was not available for study inclusion (81.5%). In 10.8% the inclusion and/or exclusion criteria were not met and 3.6% were initially treated by specialists from other departments than the emergency department. Of all patients who did not meet inclusion and/or exclusion criteria, 800 were not able to provide written informed consent, 3689 did not have public health insurance, 505 had trauma-related abdominal pain, 21 were not adults and 2799 had language barriers. Patients who presented to a study centre within the two-week implementation period between control and intervention phase were screened but inclusion stopped during this time. This was the case in 2.1% of all patients. Of all eligible patients who were approached for study participation, 1181 declined participation and 2119 were included in the study. Of enrolled patients 48.0% were enrolled during the control phase and 52.0% during the intervention phase.

Between 1st September 2021 and 30th August 2023, 45,202 patients were screened in the ten study centres, and 2119 were finally enrolled. The primary reason for non-enrollment was that no study staff was available during patient presentation to the emergency department. Recruitment process, as well as further reasons for non-enrollment are detailed in Fig. 1.

Screened and enrolled patients were well comparable with respect to sex, age, and triage category at enrollment, but enrolled patients had a 20%-points higher proportion of German nationality, presented 26%-points more frequently between 7 a.m. and 7 p.m., and had a 15%-points higher self-reported pain score than screened patients (Supplementary Table S1).

Of all enrolled patients, 1017 were included in the control group, and 1102 in the intervention group. Apart from a median age difference of four years, the group characteristics were well balanced regarding demographic and clinical parameters (Table 1). Further socio-demographic factors are shown in Supplementary Table S2. The emergency department diagnoses of all included patients as well as main hospital diagnoses of admitted patients were well comparable between groups (Supplementary Table S3a and b).

**Table 1:** Patient characteristics at baseline.

	Control group (n = 1017)	Intervention group (n = 1102)
**Sex—n (%)**		
Male	457 (44.9)	464 (42.1)
Female	560 (55.1)	637 (57.8)
Diverse	0 (0.0)	1 (0.1)
**Age** (years)—median (IQR)	49 (33; 62)	45 (31; 60)
**German nationality**—n (%)	958 (94.2)	989 (89.7)
**Triage category**—n (%)		
1–3 (urgent)	725 (71.3)	825 (74.9)
4–5 (less urgent)	283 (27.8)	271 (24.6)
Direct physician contact	5 (0.5)	6 (0.5)
Unknown	4 (0.4)	0 (0.0)
**Pain score at admission**		
0–4	399 (39.2)	445 (40.4)
5–10	475 (46.7)	533 (48.4)
Unknown	143 (14.1)	124 (11.3)
**Time of admission**—n (%)		
7 a.m.–7 p.m.	916 (90.1)	1038 (94.2)
7 p.m.–7 a.m.	101 (9.9)	64 (5.8)
Weekday (Mo-Fri)	989 (97.2)	1064 (96.6)
Weekend (Sat-Sun)	28 (2.8)	38 (3.4)
**Chronic conditions**—n (valid%)		
Previous abdominal surgery (nmiss = 172)	326/914 (35.7)	368/1033 (35.6)
Gallstones (nmiss = 176)	142/911 (15.6)	184/1032 (17.8)
Gastroesophageal reflux (nmiss = 180)	141/910 (15.5)	136/1029 (13.2)
Obstipation (nmiss = 179)	118/909 (13.0)	135/1031 (13.1)
Atrial fibrillation and flutter, cardiac		
Arrhythmia (nmiss = 172)	87/916 (9.5)	82/1031 (8.0)
Diabetes mellitus (nmiss = 169)	88/918 (9.6)	81/1032 (7.8)
Diverticulosis (nmiss = 174)	83/914 (9.1)	83/1031 (8.1)
Lactose intolerance (nmiss = 180)	70/907 (7.7)	96/1032 (9.3)
Hepatitis (nmiss = 173)	39/913 (4.3)	33/1033 (3.2)
Bowel syndrome (nmiss = 180)	66/908 (7.3)	78/1031 (7.6)
Kidney stones (nmiss = 181)	68/908 (7.5)	58/1030 (5.6)
Malignant tumor (nmiss = 180)	67/907 (7.4)	68/1032 (6.6)
Chronic inflammatory intestinal disease (nmiss = 169)	53/916 (5.8)	61/1034 (5.9)
Peripheral arterial occlusive disease (nmiss = 183)	30/908 (3.3)	23/1028 (2.2)
Endometriosis (nmiss = 182)	20/909 (2.2)	23/1028 (2.2)
**Risk factors**—n (valid%)		
Hypertension (nmiss = 194)	281/909 (30.9)	269/1016 (26.5)
Smoking (nmiss = 178)	of 921	of 1020
Daily	208 (22.6)	216 (21.2)
Sometimes	86 (9.3)	83 (8.1)
Previously	297 (32.2)	319 (31.3)
Never	330 (35.8)	402 (39.4)
Hyperlipidemia (nmiss = 245)	162/878 (18.5)	186/996 (18.7)
**Abdominal pain onset**—n (valid%) (nmiss = 170)	of 926	of 1023
<1 h	41 (4.4)	39 (3.8)
1–6 h	135 (14.6)	137 (13.4)
6–24 h	169 (18.3)	209 (20.4)
24 h to <1 week	303 (32.7)	364 (35.6)
1 week or longer	278 (30.0)	274 (26.8)
**Frequency of abdominal pain episodes**—n (valid%) (nmiss = 168)	of 927	of 1024
Once	225 (24.3)	251 (24.5)
More than once	702 (75.7)	773 (75.5)

Triage category is the urgency category assigned to the patients by triage nurses at admission to the emergency department. Of the ten study centres 5 used the Manchester Triage System and 5 used the Emergency Severity Index. The emergency department triage results in five categories: 1—resuscitation, 2—emergent, 3—urgent, 4—less urgent, 5—non-urgent. The pain score at admission was assessed by a numeric rating scale from 0 to 10; Abbreviations: IQR—inter quartile ranges; n—number; nmiss—number of missing values.

### Protocol deviations

In the control group, 10.4% of patients were lost to 30-day follow-up (n = 106) as opposed to 7.8% in the intervention group (n = 86). The primary endpoints were available in over 90% of the study population (Supplementary Table S4). The most common protocol deviation regarding the intervention process was a missing ECG at admission (33.2%) which is mostly due to physician overruling in lower abdominal and pelvic pain (Supplementary Table S5).

### Primary outcomes

**Fig. 2:**
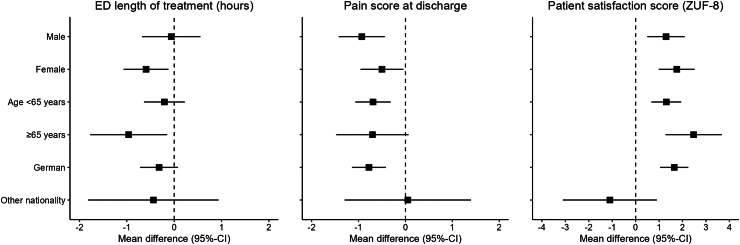
Exploratory subgroup analyses of the primary study endpoints in subgroups of sex, age and nationality. Shown are mean differences between intervention and control group with 95% confidence intervals.

Emergency department length of treatment did not decrease significantly (mean adjusted difference (model I): −0.31 h; 95% confidence interval (CI) (−0.70 to 0.07); p = 0.11) while pain score and patient satisfaction improved significantly with a mean adjusted difference of −0.69 (95% CI −1.04 to −0.34; p = 0.0002) and 1.54 (95% CI 0.96 to 2.12; p < 0.00001) respectively. Additional adjustment for age (model II) led to minor changes in outcome estimates (Table 2). Recruitment numbers and mean values of the three primary outcomes by centre and time steps are additionally illustrated in Fig. 2 and Supplementary Fig. S2C and 3A. Exploratory subgroup analyses of the primary endpoints indicated potential differences regarding emergency department length of treatment in association with sex, age, and nationality, while only nationality might have affected pain and patient satisfaction scores (Fig. 2 and Supplementary Table S6). In the per-protocol analyses 93.7% of patients (n = 953) were included in the control group and 44.1% (n = 486) in the intervention group (Supplementary Table S7). The difference of emergency department length of treatment was again not significant in the per-protocol analysis (mean adjusted difference (model 1): −0.21 (95% CI −0.72 to 0.29; p = 0.41) while the effect was slightly more pronounced regarding pain score (−0.86 (95% CI −1.29 to −0.43; p = 0.0001) and patient satisfaction (1.81 (95% CI 1.11 to 2.50; p < 0.0001), Supplementary Table S8).

**Table 2:** Primary endpoints.

Primary endpoint	Control group (n = 1017)	Intervention group (n = 1102)	Model	Mean group difference (95%CI)	p-value
Emergency department length of treatment (hours)	Mean (±SD) 5.2 ± 3.0 h	Mean (±SD) 4.3 ± 2.2 h	crude	−0.95 (−1.18; −0.72)	<0.0001
			I	−0.31 (−0.70; 0.07)	0.11
			II	−0.30 (−0.68; 0.08)	0.13
Pain score at discharge	Mean (±SD) 4.3 ± 2.5	Mean (±SD) 3.6 ± 2.4	crude	−0.67 (−0.88; 0.46)	<0.0001
			I	−0.69 (−1.04; −0.34)	0.0002
			II	−0.70 (−1.05; −0.34)	0.0001
Patient satisfaction score at discharge	Mean (±SD) 26.7 ± 4.0	Mean (±SD) 27.9 ± 3.8	crude	1.23 (0.89; 1.58)	<0.0001
			I	1.54 (0.96; 2.12)	<0.0001
			II	1.55 (0.97; 2.13)	<0.0001

Results of the unadjusted comparison (crude), generalised linear mixed effects models including fixed effects for time (4-months intervals) and random effects for centre (model I) and additionally including fixed effects for age (model II).

### Secondary exploratory outcomes

The exploratory safety endpoints indicate an increase in patient safety with a reduction in 30-day mortality (intervention group 2.3%; control group 0.8%) and reduced re-presentation to the emergency department and re-admission to hospital (Table 3). A further description of patients who died in both groups is shown in Supplementary Table S9. In the control group 9.2% had an observation period in the emergency department (n = 94) as opposed to 7.2% in the intervention group (n = 79). Furthermore, there were changes in diagnostic procedures: Of note, the performance of X-ray scans was reduced by the new treatment process while ultrasound and urinary examinations were increased (Table 3) and all process times decreased (Supplementary Table S10).

**Table 3:** Exploratory data on safety, quality of care and process quality outcomes.

	Control Group (n = 1017)	Intervention group (n = 1102)	Group difference units or % (95%-CI)
**Exploratory safety endpoints–n/N (%)**			
Mortality t0	11/1017 (1.1)	5/1102 (0.4)	−0.6 (−1.4; 0.2)
Mortality t1 additional to t0 (nmiss = 5; n died t0 = 16)	12/1002 (1.2)	4/1096 (0.4)	−0.8 (−1.6; −0.03)^a^
Mortality total	23/1017 (2.3)	9/1102 (0.8)	−1.4 (−2.5; −0.4)^a^
Life-threatening conditions t0	4/1017 (0.4)	0/1102 (0)	−0.4 (−0.9; 0.1)
Life-threatening conditions t1 (nmiss = 32)	1/1002 (0.1)	4/1078 (0.4)	0.3 (−0.2; 0.8)
Life-threatening total	5/1017 (0.5)	4/1102 (0.4)	−0.1 (−0.8; 0.5)
Unplanned or prolonged hospitalization t0	2/1017 (0.2)	2/1102 (0.2)	−0.0 (−0.5; 0.4)
Unplanned or prolonged hospitalization t1 (nmiss = 32)	23/1002 (2.3)	27/1078 (2.5)	0.2 (−1.1; 1.5)
Unplanned or prolonged hospitalization total	25/1017 (2.5)	28/1102 (2.5)	0.1 (−1.3; 1.4)
Disability or permanent damage t0 (nmiss = 2)	0/1015 (0)	0/1102 (0)	–
Disability or permanent damage t1 (nmiss = 31)	0/1002 (0)	0/1079 (0)	–
Congenital anomaly or birth defect t0	0/1017 (0)	0/1102 (0)	–
Congenital anomaly or birth defect t1 (nmiss = 32)	0/1001 (0)	0/1079 (0)	–
Delir in patients aged >65 years t0	1/197 (0.5)	0/198 (0)	−0.5 (−2.2; 1.2)
Transfer to ward (nmiss = 1)	462/1016 (45.5)	504/1102 (45.7)	0.3 (−4.0; 4.5)
Stay at intensive care unit t0 (nmiss = 26)	of 1001	of 1092	
None	978 (97.7)	1072 (98.2)	−0.5 (−1.7; 0.8)
Single time	21 (2.1)	18 (1.6)	[single + multiple]
Multiple times	2 (0.2)	2 (0.2)	
Duration of stay (days), median (IQR)	4 (1; 11)	2 (1; 4)	2 (−6; 0)
**Exploratory quality of care endpoints**			
Quality of life t1, mean (±SD) (nmiss = 395)	3.69 (±0.90)	3.79 (±0.88)	0.10 (0.01; 0.18)^a^
Patient’s general satisfaction t1, mean (±SD) (nmiss = 400)	7.09 (±2.12)	7.39 (±2.11)	0.31 (0.11; 0.51)^a^
Re-presentation to emergency department t1, n (%)	81 (8.0)	68 (6.2)	1.8 (−0.5; 4.1)
Re-presentation to hospital t1, n (%)	93 (9.1)	77 (7.0)	2.2 (−0.03; 4.6)^a^
**Exploratory process quality endpoints t0**			
Frequency of specific diagnostic procedures no. (%)			
ECG (nmiss = 9)	364/1010 (36.0)	696/1100 (63.3)	27.2 (23.1; 31.3)^a^
X-ray (nmiss = 3)	84/1014 (8.3)	33/1102 (3.0)	−5.3 (−7.3; 3.3)
Sonography (nmiss = 1)	724/1016 (71.3)	951/1102 (86.3)	15.0 (11.6; 18.5)^a^
MRT (nmiss = 1)	0/1016 (0)	2/1102 (0.2)	0.2 (−0.2; 0.5)
CT (nmiss = 1)	195/1016 (19.2)	211/1102 (19.1)	−0.1 (−3.4; 3.3)
Specialist consultation (nmiss = 5)	227/1015 (22.4)	326/1099 (29.7)	7.3 (3.6; 11.0)^a^
Urinary parameters (nmiss = 5)	544/1012 (53.8)	838/1102 (76.0)	22.3 (18.3; 26.2)^a^
Blood culture (nmiss = 1)	50/1016 (4.9)	52/1102 (4.7)	−0.2 (−2.1; 1.7)
Antibiotic treatment (nmiss = 3)	119/1015 (11.7)	147/1101 (13.4)	1.6 (−1.2; 4.4)
Blood culture before antibiotic treatment	26/119 (21.8)	31/147 (21.1)	−0.8 (−10.8; 9.1)
Analgesics treatment (nmiss = 8)	487/1011 (48.2)	505/1100 (45.9)	−2.3 (−6.5; 2.0)
Opioid treatment (nmiss = 8)	50/1012 (4.9)	58/1099 (5.3)	0.3 (−1.6; 2.2)
Specific diagnoses	997/1017 (98.0)	1092/1102 (99.1)	1.1 (0.01; 2.1)^a^

Exploratory safety, quality of care, process quality outcomes t0: index emergency department and hospital stay, t1: 30 day follow-up period, nmiss: number of missing values.

a Exploratory findings that were significant (95%-CI not including the zero).

## Discussion

The investigated structured and digitally-supported care process for patients with non-traumatic abdominal pain^10^ in the emergency department led to a significant improvement of the patient reported primary endpoints pain score and patient satisfaction at the end of emergency department treatment while emergency department length of treatment did not change significantly when adjusted for centre effects. Exploratory endpoints in general were improved by the investigated treatment process. This is true for safety endpoints (i.e., mortality), diagnostic procedures and process times. The most unexpected result was the observed difference in 30-day mortality (control group: 2.3% vs. intervention group: 0.8%). The generally low mortality as compared to other studies is likely to be caused by the prospective exclusion of patients with suspected sepsis or shock due to ethical considerations. The comparison of patients who died within 30 days between control group and intervention group shows that patients who died in the intervention group are older, more frequently male and had a higher triage category. This could thus hint to prevented deaths in younger women with lower triage categories. The low mortality in the intervention group might therefore be an intervention effect but still results are exploratory in nature and should not be over-estimated. Further investigations in larger (i.e., future routine data) samples are warranted. Also, the exploratory quality of care endpoints at 30 days showed an improvement of patient quality of life and patient’s general satisfaction as well as a decrease in re-presentation to the emergency department and re-admission to hospital when comparing intervention group to control group. Regarding the execution of diagnostic procedures in the emergency department (exploratory process quality endpoints), an increase in use of ECGs, sonographies, specialist consultations, and urine examination was observed while x-ray use was reduced and CT, and MRT were comparable between both groups. The new care pathway further led to an increase in specific diagnoses. Taking all these endpoints into account it becomes clear that the overall benefit of the process in sense of efficacy will require an analysis, which includes also health care utilisation outside the emergency department. While all process times were reduced and emergency department length of treatment was not significantly different between groups even though more diagnostic procedures were applied, there is a benefit regarding general emergency department processes. The costs associated with certain diagnostic procedures in the emergency department vary between countries and therefore cost-effectiveness might differ. In Germany, reimbursement of emergency department for outpatient care is based on lump sum remuneration and only partly based on procedures in the emergency department. Thus, patients or their insurance companies are not billed for real costs, which are covered by the hospitals. It is therefore unlikely that the increased use of diagnostic procedures will lead to a significant increase in the costs of emergency care in the German health care system. Reimbursement of emergency care for admitted patients is a non-specified part of the hospital DRG. With respect to 30 days economic data, it becomes clear that intervention group patients had lower costs, mainly due to less readmissions. These data will be presented in detail elsewhere. Comparable effects have been shown for Chest Pain Units in the past with higher resource use in the emergency department/Chest Pain Unit, but overall health economic benefit.^23^

The benefits of digitisation in health care include decision and process support. In emergency medicine, the unpredictable amount and type of patients, lack of staff, and after hours care challenges lead to mistakes, which may be avoided by digitally supported clinical processes.^24^ ‘Units’ in emergency medicine have specific care processes and resources but don’t necessarily include a physical structure (e.g., stroke unit, chest pain unit). Our hypothesis was that a digitally-supported process for abdominal pain (APU-process) would lead to better patient-reported outcomes and/or a reduction of emergency department length of treatment due to more efficient, precise, and timely initiation of diagnostics and treatment.

Given that emergency department length of treatment is closely related to emergency department crowding^25^ it is important to note that emergency department length of treatment remained stable even though a more detailed process was introduced, and a negative impact on crowding is therefore unlikely.

The significant improvement of patient reported outcomes indicates that the intervention led to an improved care process: The standardised and repeated pain assessment and administration of analgesics led to a significant pain reduction, and treatment satisfaction in general was improved as well.^18^

The analysis of exploratory endpoints showed that the intervention process was safe and could be hypothesised to reduce mortality (2.3% vs. 0.8%) while it might be also more efficient in the long-term with a reduced rate of re-presentation to the emergency department (8.1% vs. 6.1%) and re-admission within 30 days (9.2% vs. 6.9%). A high re-admission rate to the emergency department was also identified as a common problem in patients with abdominal pain in other studies with in general comparable admission rates (6–10%).^4^^,^^26^ Of note, the proportion of patients with specific diagnoses was comparably high in our study with a proportion of only 2% non-specific abdominal pain diagnoses already in the control group. Other studies showed proportions around 30%.^2^^,^^4^ This might be attributed to the study setting in general. Even though physicians were not trained in the APU-process during the control period, they were trained in all study procedures in general and well aware that included cases were thoroughly investigated which might have led to a more detailed documentation with more specific diagnostic codes. An important step in the APU-process is the early conduction of abdominal ultrasound by emergency department physicians. The usefulness of an early ultrasound by emergency department physicians in the work-up of abdominal pain patients in the emergency department has already been illustrated by other studies which showed an improvement of diagnostic accuracy and a decrease in further radiographic and laboratory testing.^27^ This is also clearly reflected in the frequency of diagnostic procedures in our study with a reduction of performed X-rays of 5% and an increase of abdominal ultrasound of 15%.

The subgroup analyses indicated age and sex differences in emergency department length of treatment. Age-related differences in the characteristics of patients with non-traumatic abdominal pain have been previously reported and might be causes for the observed differences in our study.^4^^,^^6^^,^^28^^,^^29^ The same applies to sex differences.^3^^,^^5^^,^^6^ Regarding nationality the subgroups with non-German patients were small which led to lower precision in estimates, but the results still indicated differences which have also been reported for migration previously and warrant further investigation.^3^ All results regarding secondary endpoints and subgroup analyses are exploratory in nature and should be regarded as hypothesis-generating for future trials.

The generalisability of our results to the whole population of patients with non-traumatic abdominal pain is restricted with regard to presentation time, citizenship, and initial pain score since differences were observed between screened and included patients regarding these variables. In our study, we could only include patients with sufficient German language skills to obtain written informed consent and to assess patient reported information which has led to selection bias. A broader implementation of the APU-process should therefore be closely monitored in this specific subgroup. Furthermore, patients with high suspicion of sepsis and shock were not eligible for participation, and therefore, mortality was in general lower when compared to routine data analyses.^1^^,^^5^^,^^6^ In the intervention process a deterioration of patient status is monitored frequently, which might also be reflected by lower mortality in the intervention group.^10^ Therefore, it seems likely that patients at higher risk of sepsis or shock would benefit from the APU-process as well. Since the implementation of the APU process required a specific training of the treating physicians an individual randomisation and blinding was not possible, but specific adjustments were integrated in the analysis of the stepped wedge trial. While we measured the effects of the intervention on the study population it remains questionable how other emergency department patients were affected by the APU-process. It cannot be excluded that other patients experienced longer waiting times, and resources were driven away from them because of the more standardised treatment of abdominal pain patients. Such effects would also need to be closely monitored while implementing the new APU-process in the emergency department. Pregnant women were not excluded from participation by design but according to the final study documentations no pregnant patient was included either. Still the follow-up period wouldn’t have been long enough to assess congenital anomalies or birth defects as part of the serious adverse events-criteria. These events were not expected given the character of the intervention but should still be monitored given a routine implementation of the intervention. Loss to 30-day follow-up differed between control group and intervention group. Although this finding is unlikely to be related to study procedures and losses can be assumed to be random, patient satisfaction may have been a potential confounder and therefore an effect cannot be completely excluded. However, this effect did not influence the primary endpoints, since the primary endpoints were assessed at the end of treatment in the ED. Missing values for primary endpoints occurred mainly because patients left the ED without waiting for primary endpoint assessment, forgot to fill in the questionnaire, or because the treating physicians forgot to hand out the questionnaire to the patients in the first place. We therefore believe that these missing values can be considered as missing at random as well. Finally, performance bias in the control group cannot be excluded since blinding was not possible due to the intervention of the study.

The APU treatment process could be implemented using either the flow chart (Supplementary Fig. S1) or digitised support. Both applications are valid for routine clinical implementation. Adherence to the protocol is expected to be higher with a digitised implementation, as certain steps in the process can be digitally checked and the emergency physician is therefore given more guidance. Our recommendation would be to implement the APU process digitally in the hospital's existing Patient Data Management System, as this would allow for simultaneous documentation of each step and improvement of processes and treatment. The standard operating procedures for each treatment step could be adapted to meet the specific needs of each emergency department.

### Conclusions

The new digitally-supported treatment process for patients with non-traumatic abdominal pain in the ED significantly improved patient reported outcomes while emergency department length of treatment remained stable. Exploratory outcomes further hint to an improved efficiency of treatment and higher patient safety including reduced emergency department re-admission, re-hospitalisation, and mortality. This improvement in exploratory outcomes was associated with an increased use of diagnostic resources in the emergency department. Implementation of the APU-process should therefore be carefully considered from a health economic perspective, and its implementation should be monitored in terms of resource use in routine clinical routine conditions.

## Contributors

All authors made substantial contributions to conception and design; and/or acquisition, and/or analysis or interpretation of the data. KV, DH, JF, KV, AFR and YNW accessed and analysed the data presented in this manuscript. AS and MM wrote the original draft of the article and all authors provided a critical revision and editing of the manuscript and approve the final version to be published. All authors agree to be responsible for all aspects of the work.

## Data sharing statement

The original study data cannot be made available due to data protection requirements but the study protocol, statistical analysis plan and informed consent forms can be made available from the corresponding author upon request.

## Declaration of interests

For this study, the authors received funding from the Innovations Fund of the German Federal Joint Committee (G-BA) under the grant number 01NVF19025. In addition, the working group of AS and MM received financial support from various German public funding sources (BMBF, BMG, Innovationsfonds, NUM), Roche Diagnostics as well as the German Research Foundation. AS also received consulting fees from the Zentralinstitut für Kassenärztliche Versorgung (Zi) and from the Federal Government (Bundestag) for work unrelated to the present manuscript. In this context, she provided expert testimony for the Bundestag, likewise independent of the content of this publication. LS serves as Deputy Head of Department “Öffentliche Gesundheit und Public Health”, is a member of the extended board of the German Society for Social Medicine and Prevention (DGSMP) and acts as a spokesperson of the DGSMP working group “Migration and Health”, all on a voluntary basis. LS is also a member of the German Society of Sociology. Unrelated to this manuscript, HD gave a lecture/presentation titled “Rescue Cases” on behalf of AstraZeneca. He further holds a leadership position as a scientific director at INOB and as vice president of DGINA.

AFR received a reduced participation fee for her active participation (poster presentation) at the DIVI 2024. Both AFR and BS are members of the DGINA e.V. In addition, BS is a member of the DGIM e.V. FI is employed by Charité–Universitätsmedizin Berlin. KV received financial support for attending meetings or travel from the German Innovationsfonds.

Furthermore, AW is a member of der German Society of Sociology. MM has provided consultancy services to Thermofisher and Roche Diagnostics outside the scope of this manuscript. He has also delivered lectures/presentations for Diasorin, Roche Diagnostics, AstraZeneca, Sanofi, EMCREG and PeerVoice. MM is a member of the Chair of the EUSEM Research Committee and an expert panel member of the Research Institute of the Local Health Care Funds (WIdO).
