# Supplementary material for: Management of non-traumatic abdominal pain in the emergency department: a multicentre, stepped-wedge, cluster-randomised trial
Source: Lancet Reg Health Eur. 2025 Jun 26;55:101362. doi: 10.1016/j.lanepe.2025.101362 (PMC12268095; doi:10.1016/j.lanepe.2025.101362)
Supplement: Supplementary Figs. S1–S3 and Tables S1–S10 [file mmc1.docx]

**Supplementary Appendix**

Supplement to:

Management of non-traumatic abdominal pain in the emergency department:

a multicentre, stepped-wedge, cluster-randomised trial

*Anna Slagman, Ph.D; Liane Schenk, Ph.D.; Dörte Huscher, Ph.D.; Lisa Arnold ,MSc; Harald Dormann, Ph.D.; Johannes Drepper, Ph.D.; Larissa Eienbröker ,MSc; Antje Fischer-Rosinsky, Ph.D.; Johann Frick, MPH; Lukas Helbig, M.D.; Dirk Horenkamp-Sonntag, Ph.D.; Freddy Irorutola, M.D.; Tim Klinge, M.D.; Thomas Reinhold, Ph.D.; Peter Schily, M.D.; Britta Stier, M.D.; Katharina Verleger, MPH; Andreas Wagenknecht,Ph.D.; Yves Noel Wu, MSc; Martin Möckel, Ph.D.*

**Table of Content**

[**Supplemental Methods** 3](#_Toc197269004)

[**The Abdominal Pain Unit (APU) treatment process** 3](#_Toc197269005)

[**Supplemental Results** 5](#_Toc197269006)

[**Table S1: Comparison of the ED populations screened for participation in the APU-study, eligible for study participation and the finally included study population who provided written informed consent.** 5](#_Toc197269007)

[**Table S2: Socio-demographic characterization of the study population** 5](#_Toc197269008)

[**Table S3a: Top 20 diagnoses in the emergency department** 6](#_Toc197269009)

[**Table S3b: Top 10 main hospital diagnoses at discharge of all patient admitted to hospital ward** 7](#_Toc197269010)

[**Table S4: Primary Outcomes availability** 7](#_Toc197269011)

[**Figure S2: Recruitment numbers by center and study group** 8](#_Toc197269012)

[**Figures S3a-c: Mean values of the three primary outcomes by center, time step and study group** 9](#_Toc197269013)

[**Table S5: Protocol Deviations** 10](#_Toc197269014)

[**Table S6: Exploratory subgroup analyses of the primary endpoints** 11](#_Toc197269015)

[**Table S7: Patient Characteristics of the per-protocol population at Baseline** 11](#_Toc197269016)

[**Table S8: Primary Endpoints, per-protocol analysis** 12](#_Toc197269017)

[**Table S9: Characteristics of patients who died within 30 days of ED treatment** 12](#_Toc197269018)

# **Supplemental Methods**

## **The Abdominal Pain Unit (APU) treatment process**

**Figure S1: Detailed flow chart of the APU treatment process**


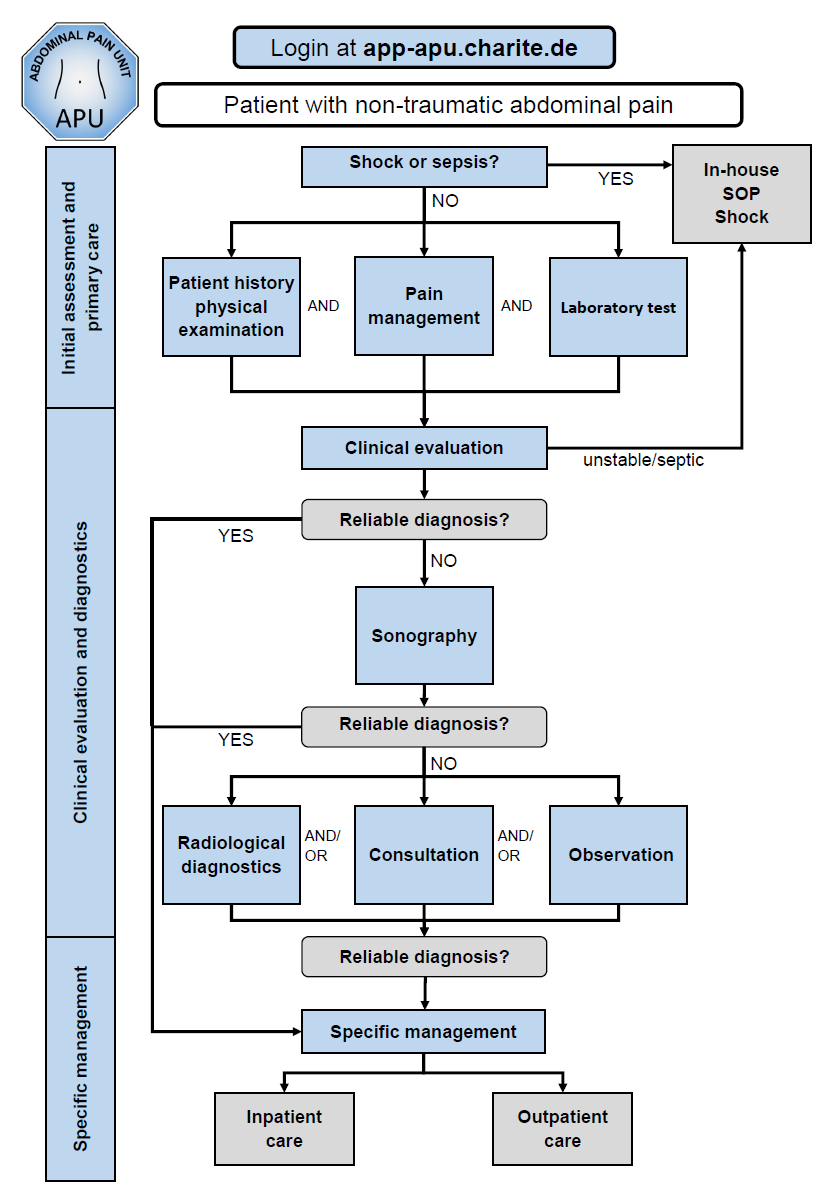


*Legend Figure S1: In this figure the intervention, consisting of a standardized, digitally-supported treatment pathway (APU-process) for patients with non-traumatic abdominal pain is depicted. Each step could be specified by hospital specific standard operating procedures (SOPs).*

# **Supplemental Results**

## **Table S1: Comparison of the ED populations screened for participation in the APU-study, eligible for study participation and the finally included study population who provided written informed consent.**

|  |  | **Screened (n=45,202)** | **Eligible (n=3,300)** | **With consent (n=2,119)** |
| --- | --- | --- | --- | --- |
| **Sex n (%)** | Male | 19,659 (43·5) | 1,430 (43·3) | 921 (43·5) |
|  | Female | 25,533 (56·5) | 1,869 (56·6) | 1,197 (56·5) |
|  | diverse | 3 (0) | 1 (0) | 1 (0) |
|  | unknown | 7 (0) | (0) | (0) |
| **Age (years)** | Mean±SD | 50·4 ± 20·6 | 49·2 ± 19·5 | 47·5 ± 18·2 |
| **Nationality**  **n (%)** | Germany  other  unknown | 32,153 (71·1)  6,970 (15·4)  6,079 (13·4) | 2,920 (88·5)  248 (7·5)  132 (4·0) | 1,947 (91·9)  149 (7·0)  23 (1·1) |
| **Time of admission**  **n (%)** | 7 am – 7 pm | 30,159 (66·7) | 2,957 (89·6) | 1,954 (92·2) |
|  | 7 pm – 7 am | 14,993 (33·2) | 343 (10·4) | 165 (7·8) |
|  | unknown | 50 (0·1) | (0) | (0) |
| **Triage category n (%)** | 1-3 (urgent) | 33,363 (73·8) | 2,260 (68·5) | 1,550 (73·1) |
|  | 4-5 (less urgent) | 10,935 (24·2) | 1,014 (30·7) | 554 (26·1) |
|  | direct physician contact | 548 (1·2) | 18 (0·5) | 11 (0·5) |
|  | unknown | 356 (0·8) | 8 (0·2) | 4 (0·2) |
| **Pain score at admission n (%)** | 0 - 4 | 14,398 (31·9) | 1,260 (38·2) | 844 (39·8) |
|  | 5 - 10 | 14,554 (32·2) | 1,446 (43·8) | 1,008 (47·6) |
|  | unknown | 16,250 (35·9) | 594 (18) | 267 (12·6) |

Legend Table S1: Demographic and clinical characteristics of patients screened for participation in the APU-study, those eligible for participation and of the final study population. Abbreviations: n=total number

## **Table S2: Socio-demographic characterization of the study population**

|  | **Control**  **group**  **(n=1,017)** | **Intervention**  **group**  **(n=1,102)** |
| --- | --- | --- |
| **Relationship status, n (%)** |  |  |
| Partnered | 572 (56·2) | 633 (57·4) |
| Unpartnered | 350 (34·4) | 394 (35·8) |
| Missing | 95 (9·3) | 75 (6·8) |
| **Household size, n (%)** |  |  |
| Single | 245 (24·1) | 267 (24·2) |
| Two or more persons | 668 (65·7) | 752 (68·2) |
| Missing | 104 (10·2) | 83 (7·5) |
| **In need of care, n (%)** |  |  |
| Yes | 63 (6·2) | 65 (5·9) |
| No | 856 (84·2) | 963 (87·4) |
| Missing | 98 (9·6) | 74 (6·7) |
| **Financial situation, n (%)** |  |  |
| Very good | 104 (10·2) | 132 (12·0) |
| Good | 444 (43·7) | 545 (49·5) |
| Fair | 262 (25·8) | 259 (23·5) |
| Difficult | 69 (6·8) | 57 (5·2) |
| Very difficult | 23 (2·3) | 21 (1·9) |
| Missing | 115 (11·3) | 88 (8·0) |
| **Migration context, n (%)** |  |  |
| 1^st^ generation | 110 (10·8) | 172 (15·6) |
| 2^nd^ generation | 53 (5·2) | 64 (5·8) |
| None | 748 (73·5) | 785 (71·2) |
| Missing | 106 (10·4) | 81 (7·4) |
| **Social support** **n (%)** |  |  |
| Poor | 160 (15·7) | 145 (13·2) |
| Moderate | 465 (45·7) | 500 (45·4) |
| Strong | 278 (27·3) | 372 (33·8) |
| Missing | 114 (11·2) | 85 (7·7) |
| **Educational Classification n (%)** |  |  |
| Low | 184 (18·1) | 264 (24·0) |
| Medium | 506 (49·8) | 491 (44.6) |
| High | 197 (19·4) | 255 (23·1) |
| Missing | 130 (12·8) | 92 (8·3) |

Legend Table S2: Sozio-demographic characteristics of the study population in comparison of control and intervention group. Abbreviations: n=total number

## **Table S3a: Top 20 diagnoses in the emergency department**

| **Diagnoses control group**  **(n=1,119)** | | | | **Diagnoses intervention group**  **(n=1,075)** | | | |
| --- | --- | --- | --- | --- | --- | --- | --- |
| **ICD_10 code** | **Diagnosis text** | **n** | **%** | **ICD_10 code** | **Diagnosis text** | **n** | **%** |
| R10 | Abdominal and pelvic pain | 309 | 27·6% | R10 | Abdominal and pelvic pain | 391 | 36·4% |
| K80 | Cholelithiasis | 57 | 5·1% | K35 | Acute appendicitis | 52 | 4·8% |
| K57 | Diverticular disease of intestine | 53 | 4·7% | K80 | Cholelithiasis | 50 | 4·7% |
| A09 | Infectious gastroenteritis and colitis, unspecified | 48 | 4·3% | K57 | Diverticular disease of intestine | 49 | 4·6% |
| K35 | Acute appendicitis | 46 | 4·1% | K29 | Gastritis and duodenitis | 48 | 4·5% |
| K29 | Gastritis and duodenitis | 32 | 2·9% | A09 | Infectious gastroenteritis and colitis, unspecified | 40 | 3·7% |
| K56 | Paralytic ileus and intestinal obstruction without hernia | 31 | 2·8% | K56 | Paralytic ileus and intestinal obstruction without hernia | 33 | 3·1% |
| K85 | Acute pancreatitis | 30 | 2·7% | K85 | Acute pancreatitis | 32 | 3·0% |
| K83 | Other diseases of biliary tract | 22 | 2·0% | N39 | Other disorders of urinary system | 18 | 1·7% |
| K59 | Other functional intestinal disorders | 19 | 1·7% | K81 | Cholecystitis | 17 | 1·6% |
| K81 | Cholecystitis | 17 | 1·5% | K92 | Other diseases of digestive system | 16 | 1·5% |
| R11 | Nausea and vomiting | 16 | 1·4% | N20 | Calculus of kidney and ureter | 15 | 1·4% |
| U99 | Special procedures for coding purposes | 16 | 1·4% | R11 | Nausea and vomiting | 15 | 1·4% |
| N20 | Calculus of kidney and ureter | 14 | 1·3% | K50 | Crohn’s disease | 12 | 1·1% |
| N39 | Other disorders of urinary system | 13 | 1·2% | N83 | Noninflammatory disorders of ovary, fallopian tube and broad ligament | 12 | 1·1% |
| U07 | Diseases with unclear aetiology, assigned and unassigned key numbers (wahrscheinlich COVID-19, U07.1 positiv / U07.2 negativ) | 12 | 1·1% | K83 | Other diseases of biliary tract | 11 | 1·0% |
| E87 | Other disorders of fluid, electrolyte and acid-base balance | 9 | 0·8% | K51 | Ulcerative colitis | 9 | 0·8% |
| K76 | Other diseases of liver | 9 | 0·8% | K59 | Other functional intestinal disorders | 9 | 0·8% |
| N83 | Noninflammatory disorders of ovary, fallopian tube and broad ligament | 8 | 0·7% | N13 | Obstructive and reflux uropathy | 8 | 0·7% |
| K43 | Ventral hernia | 7 | 0·6% | K86 | Other diseases of pancreas | 7 | 0·7% |

Legend Table S3a: Top 20 diagnoses in the ED of all patients (admitted and non-admitted). Multiple diagnoses per patient are possible. Abbreviations: n=total number

## **Table S3b: Top 10 main hospital diagnoses at discharge of all patient admitted to hospital ward**

| **Diagnoses control group***  **(n=525)** | | | | **Diagnoses intervention group***  **(n=563)** | | | |
| --- | --- | --- | --- | --- | --- | --- | --- |
| **ICD_10 code** | **Diagnosis text** | **n** | **%** | **ICD_10 code** | **Diagnosis text** | **n** | **%** |
| K35 | Acute Appendicitis | 56 | 10·7% | K35 | Acute Appendicitis | 68 | 12·1% |
| K57 | Diverticular disease of the intestine | 46 | 8·8% | K80 | Cholelithiasis | 60 | 10·7% |
| K80 | Cholelithiasis | 39 | 7·4% | R10 | Abdominal and pelvic pain | 45 | 8·0% |
| K85 | Acute pancreatitis | 31 | 5·9% | K57 | Diverticular disease of the intestine | 42 | 7·5% |
| R10 | Abdominal and pelvic pain | 27 | 5·1% | K85 | Acute pancreatitis | 29 | 5·2% |
| K56 | Paralytic ileus and intestinal obstruction without hernia | 23 | 4·4% | K56 | Paralytic ileus and intestinal obstruction without hernia | 24 | 4·3% |
| K81 | Cholecystitis | 18 | 3·4% | A09 | Other and unspecified gastroenteritis and colitis of infectious and unspecified origin | 17 | 3·0% |
| K29 | Gastritis and duodenitis | 14 | 2·7% | K29 | Gastritis and duodenitis | 16 | 2·8% |
| A09 | Other and unspecified gastroenteritis and colitis of infectious and unspecified origin | 9 | 1·7% | K81 | Cholecystitis | 11 | 2·0% |
| C18 | Malignant neoplasm of the colon | 9 | 1·7% | K92 | Other diseases of digestive system | 10 | 1·8% |

Legend Table S3b: Top 10 main hospital diagnoses at discharge of admitted patients only. *nmiss control group (n=88) nmiss intervention group (n=73); Abbreviations: n=total number, nmiss= total number of missing values

##

## **Table S4: Primary Outcomes availability**

|  | **Total**  **(n=2,119)** | **Control**  **(n=1,017)** | **Intervention**  **(n=1,102)** |
| --- | --- | --- | --- |
| Length of ED treatment (h) | 2,041 (96·3) | 959 (94·3) | 1,082 (98·2) |
| Pain score at discharge | 1,962 (92·6) | 931 (91·5) | 1,031 (93·6) |
| Patient satisfaction score at discharge | 1,967 (92·8) | 935 (91·9) | 1,032 (93·6) |

Legend Table S4: Availability of all three primary endpoint in the final study population and within the control and intervention group. Abbreviations: n=total number, h=hours

## **Figure S2: Recruitment numbers by center and study group**


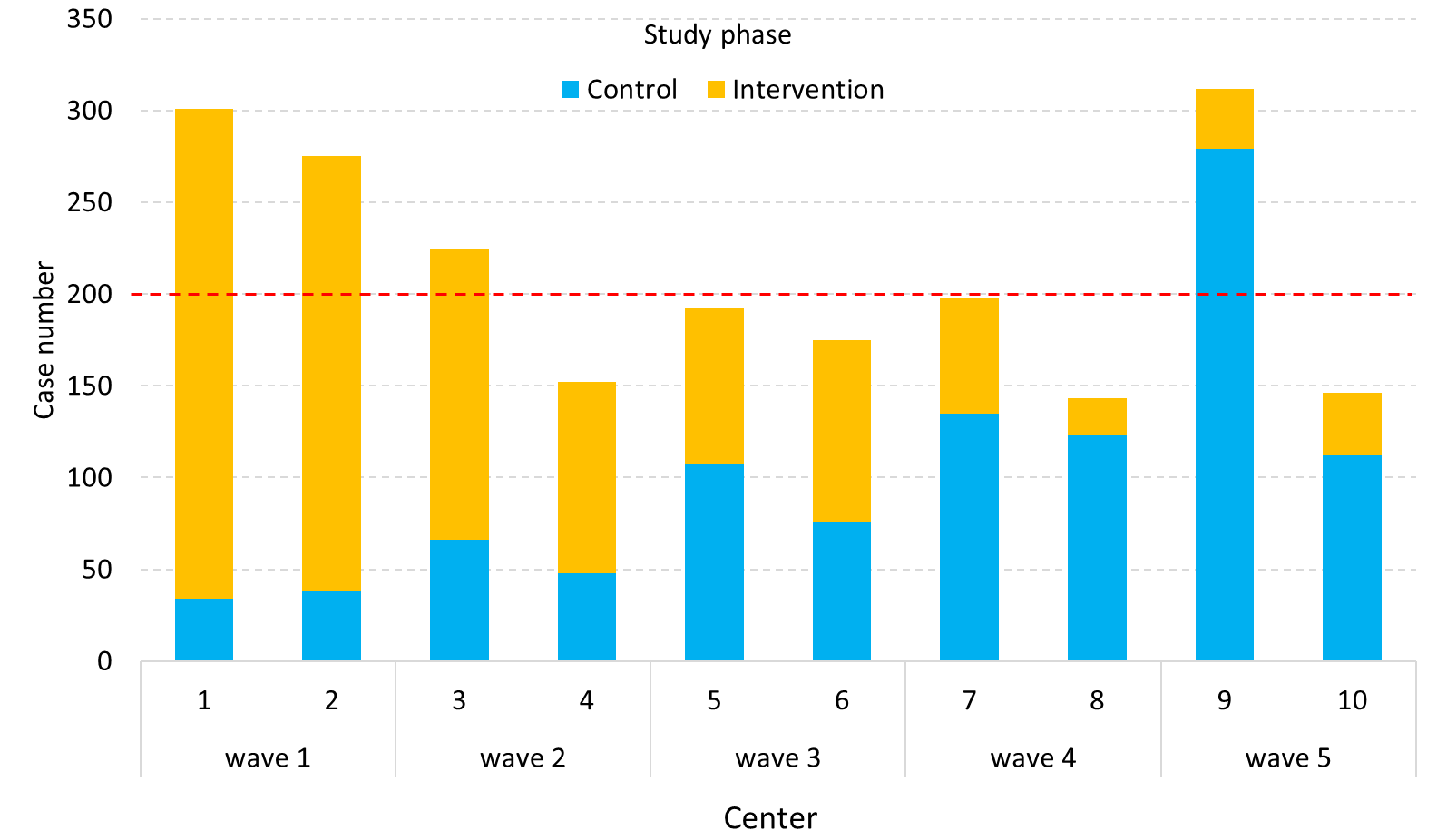

Legend Figure S2: Recruitment by centre, planned recruitment is indicated by the red dashed line. To achieve the intended sample size, centres of waves 1 and 2 as well as one centre of wave 5 were encouraged to recruit further intervention or control patients after reaching their recruitment targets.

## **Figures S3a-c: Mean values of the three primary outcomes by center, time step and study group**


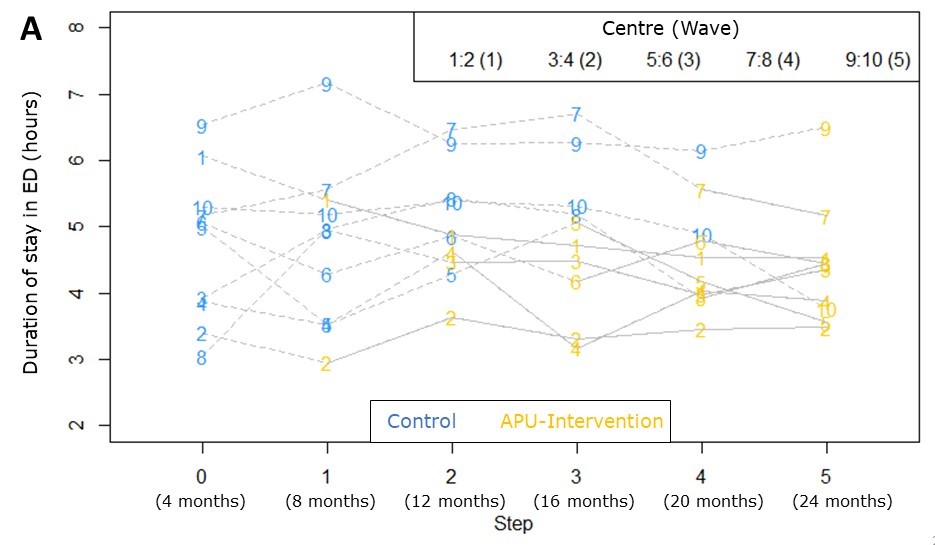


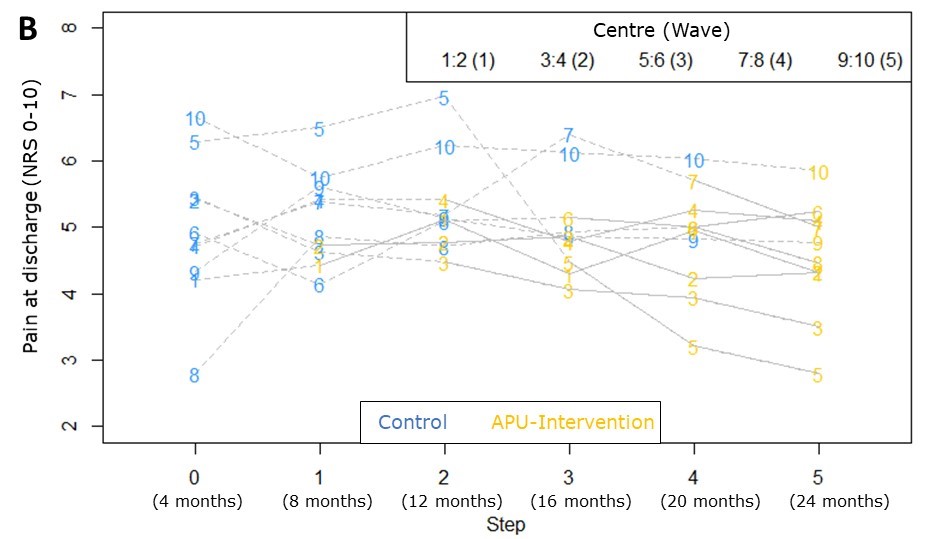


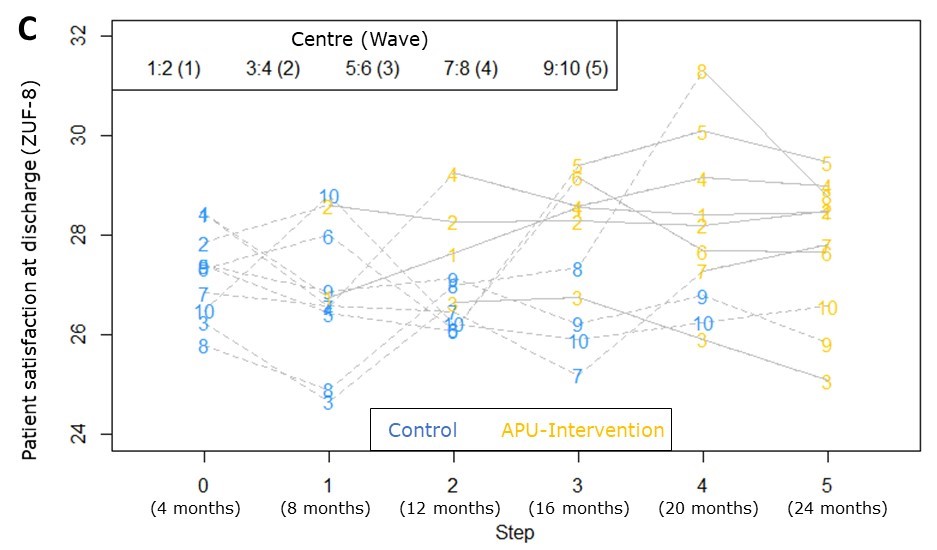


Legend Figures S3a-c: Mean values of the three primary outcomes by center and time step with colour-indicated control (blue) or intervention (yellow) phase for A) duration of treatment in the EU, B) pain score at discharge (score range 0-10), and C) patient satisfaction (score range 8-32)

## **Table S5: Protocol Deviations**

| *All data are given in absolute and relative frequencies*  *n (%)* | **Control group**  **n=1,017** | **Intervention group**  **n=1,102** |
| --- | --- | --- |
| **Lost to 30-day Follow-up^1^** | 106 (10·4) | 86 (7·8) |
| **The patient survey was not conducted at the 30day Follow-up^2^** | 202 (19·9) | 191 (17·2) |
| **Patient leaves ED against doctor’s order^3^** | 13 (1·3) | 20 (1·8) |
| **Pain score not assessed^4^** | 54 (5·3) | 66 (6·0) / 61 (5·5)* |
| **No sonography prior to CT^4^** | n.a. | 13 (1·2) / 7 (0·6)* |
| **Abdominal X-ray^4^** | n.a. | 9 (0·8) / 0 (0·0)* |
| **No ECG^4^**  **Reason “no ECG”: lower abdominal and pelvic pain**  **Other reasons** | n.a. | 366 (33·2)  270 (24·5)  96 (8·7) / 75 (6·8)* |
| **No laboratory parameters^4^** | n.a. | 2 (0·2) |
| **No blood gas analysis^4^** | n.a. | 85 (7·7) / 64 (5·8)* |
| **No urinary examination^4^** | n.a. | 200 (18·1) / 119 (10·8)* |
| **No ß-HCG in women of childbearing age (18-55 years of age: n=440)^4^** | n.a. | 79 (18·1) / 58 (13·2)* |

Legend Table S5: The depicted endpoints were used to determe protocol deviations: ^1^ – including t1 questionnaire and civil register query; 2 – 30-day (t1) follow-up questionnaire was carried; 3 – documented protocol deviation or documented reason for leaving the ED; 4 – protocol deviation documented by study nurse; * - protocol deviations from the APU-treatment process based on extracted routine emergency department treatment data extracted to study specific electronic case report forms by study nurses. Abbreviations: ED=emergency department; n=total number; CT=computer tomography; ECG=electro cardiogram;

##

## **Table S6: Exploratory subgroup analyses of the primary endpoints**

| **Outcome** | **Subgroup** | **Mean difference between intervention and control group (95%-CI)** | **p-value** |
| --- | --- | --- | --- |
| ED length of treatment (hours) | male | -0·06 (-0·68;0·55) | 0·84 |
|  | female | -0·59 (-1·07;-0·12) | 0·014 |
|  | age <65 years | -0·21 (-0·64;0·22) | 0·35 |
|  | age >=65 years | -0·96 (-1·77;-0·15) | 0·021 |
|  | unknown | -2·63 (-6·34;1·08) | 0·14 |
|  | German | -0·32 (-0·72;0·08) | 0·12 |
|  | other nationality | -0·44 (-1·82;0·94) | 0·52 |
| Pain score at discharge | male | -0·93 (-1·42;-0·44) | 0·0004 |
|  | female | -0·5 (-0·96;-0·05) | 0·031 |
|  | age <65 years | -0·69 (-1·07;-0·31) | 0·0004 |
|  | age >=65 years | -0·7 (-1·47;0·07) | 0·073 |
|  | unknown | -1·71 (-6·31;2·89) | 0·45 |
|  | German | -0·78 (-1·14;-0·42) | <0·0001 |
|  | other nationality | 0·05 (-1·3;1·4) | 0·94 |
| Patient satisfaction score at discharge | male | 1·3 (0·5;2·09) | 0·0017 |
|  | female | 1·75 (0·99;2·52) | <0·0001 |
|  | age <65 years | 1·31 (0·67;1·94) | <0·0001 |
|  | age >=65 years | 2·47 (1·27;3·67) | <0·0001 |
|  | unknown | 4·47 (-2·33;11·27) | 0·18 |
|  | German | 1·64 (1·04;2·24) | <0·0001 |
|  | other nationality | -1·1 (-3·1;0·91) | 0·28 |

Legend Table S6: Exploratory subgroup analyses of the primary endpoints with generalized linear mixed effects models including fixed effects for time (4-months intervals) and random effects for center. Abbreviations: CI=confidence interval

##

## **Table S7: Patient Characteristics of the per-protocol population at Baseline**

|  | | | **Control**  **group**  **(n=953)** | **Intervention**  **group**  **(n=486)** |
| --- | --- | --- | --- | --- |
| **Sex** – n (%) | | | | |
| Male      Female      Diverse | | | 440 (46·2) | 210 (43·2) |
|  |  |  | 513 (53·8) | 276 (56·8) |
|  |  |  | 0 (0·0) | 0 (0·0) |
| **Age** (years) – median (IQR) | | | 50 (33;62) | 51 (35;65) |
| **German nationality** – n (%) | | 899 (94·3) | | 449 (92·6) |
| **Triage category** – n (%) | | | | |
| 1-3 (urgent) | | 679 (71·2) | | 347 (71·4) |
| 4-5 (less urgent) | | 266 (27·9) | | 135 (27·8) |
| direct physician contact | | 4 (0·4) | | 4 (0·8) |
| Unknown | | 4 (0·4) | | 0 (0·0) |
| **Pain Score at admission** | |  | |  |
| 0 – 4 | | 386 (41·0) | | 208 (43·2) |
| 5 – 10 | | 461 (48·9) | | 217 (45·1) |
| Unknown | | 95 (10·1) | | 56 (11·6) |
| **Time of admission** – n (%) | | | |  |
|  | 7 a.m. – 7 p.m. | 855 (89·7) | | 454 (93·4) |
|  | 7 p.m. – 7 a.m. | 98 (10·3) | | 32 (6·6) |
|  | weekday (Mo-Fri) | 925 (97·1) | | 472 (97·1) |
|  | weekend (Sat-Sun) | 28 (2·9) | | 14 (2·9) |

 Legend Table S7: Patient characteristics of the per protocol population at baseline; Abbreviations: n=total number; IQR=inter quartile rage

## **Table S8: Primary Endpoints, per-protocol analysis**

| **Primary endpoint** | **Control group**  **(n=953)** | **Intervention group**  **(n=486)** | **Model** | **Mean group difference (95%CI)** | **p-value** |
| --- | --- | --- | --- | --- | --- |
| **ED length of treatment (hours)** | Mean (±SD) 5·3±3·1 h | Mean (±SD)  4·3±2·2 h | crude | -0·98 (-1·26;-0·70) | <0·0001 |
|  |  |  | I | -0·21 (-0·72; 0·29) | 0·41 |
|  |  |  | II | -0·25 (-0·75; 0·26) | 0·34 |
| **Pain score at discharge** | Mean (±SD)  4·3±2·4 | Mean (±SD)  3·4±2·3 | crude | -0·89 (-1·16; -0·62) | <0·0001 |
|  |  |  | I | -0·86 (-1·29; -0·43) | 0·0001 |
|  |  |  | II | -0·85 (-1·28; -0·42) | 0·0001 |
| **Patient satisfaction score at discharge** | Mean (±SD  26·7±4·0) | Mean (±SD)  28·0±3·8 | crude | 1·35 (0·90;1·79) | <0·0001 |
|  |  |  | I | 1·81 (1·11; 2·50) | <0·0001 |
|  |  |  | II | 1·80 (1·10; 2·49) | <0·0001 |

Table S8: Primary endpoints – per-protocol analysis. Results of the unadjusted comparison (crude), generalized linear mixed effects models including fixed effects for time (4-months intervals) and random effects for center (model I) and additionally including fixed effects for age (model II).

## **Table S9: Characteristics of patients who died within 30 days of ED treatment**

|  | | **Control group**  (n=23) | | **Intervention group**  (n=9) | |
| --- | --- | --- | --- | --- | --- |
| **Age** | |  |  |  |  |
|  | **mean** | 65·9 |  | 75·9 |  |
|  | **median** | 63 |  | 78 |  |
|  | **SD** | 8·6 |  | 10·8 |  |
| **Sex** | |  |  |  |  |
|  | **Men (n/%)** | 14 | 60·9 | 6 | 66·7 |
|  | **Women (n/%)** | 9 | 39·1 | 3 | 33·3 |
| **Nationality** | |  |  |  |  |
|  | **German (n/%)** | 23 | 100 | 9 | 100 |
| **Triage** | |  |  |  |  |
|  | **1-3 (urgent) (n/%)** | 17 | 73·9 | 8 | 88·9 |
|  | **4-5 (less-urgent) (n/%)** | 5 | 21·7 | 1 | 11·1 |
|  | **Not available (n/%)** | 1 | 4·3 |  |  |
| **Pain Score** | |  |  |  |  |
|  | **5-10 (n/%)** | 13 | 56·5 | 5 | 55·6 |
|  | **0-4 (n/%)** | 8 | 34·8 | 3 | 33·3 |
|  | **Not available (n/%)** | 2 | 8·7 | 1 | 11·1 |

Legend Table S9: Characteristics of patients who died within 30 days of ED treatment; Abbreviations: n=total number

**Table S10: Process times in the ED**

|  | **Control group**  **n=1,017** | | **Intervention group**  **n=1,102** | |
| --- | --- | --- | --- | --- |
|  | **Utilisation,**  **n (%)** | **Process time (min), median (Q1-Q3)** | **Utilisation,**  **n (%)** | **Process time (min), median (Q1-Q3)** |
| **X-ray** | 84 (8·3) | 189 (132-269)  (n=82) | 33 (3·0) | 181 (110-247)  (n=32) |
| **CT** | 195 (19·2) | 252 (187-350)  (n=185) | 211 (19·1) | 228 (177-309)  (n=207) |
| **Ultrasound** | 724 (71·3) | 158 (87 - 261)  (n=488) | 951 (86·3) | 126 (72 - 200)  (n=686) |
| **Urinary examination** | 544 (53·8) | 81 (44 - 149)  (n=520) | 838 (76·0) | 73 (39 - 124)  (n=827) |
| **Consultation** | 227 (22·4) | 282 (187 - 385)  (n=205) | 326 (29·7) | 225 (147 - 319)  (n=301) |
| **Antibiotic treatment** | 119 (11·7) | 235 (159 - 348)  (n=80) | 147 (13·4) | 208 (140 - 329)  (n=133) |
| **Analgesic treatment with opioids** | 50 (4·9) | 140 (80 - 202)  (n=38) | 58 (5·3) | 91 (52 - 193)  (n=51) |

Table S10: Process times (minutes) between administrative registration in the ED and diagnostic and therapeutic measures in the ED. Plausibility cut-off was set at a minimum of 5 minutes between administrative ED registration and any other procedure and to a maximum of 720 minutes. Abbreviations: CT=computer tomography; min=minutes; n=absolute number; Q1=1^st^ quartile; Q3=3^rd^ quartile

| Section/Topic | Item No | Checklist item | Reported on page No |
| --- | --- | --- | --- |
| Title and abstract | | | |
|  | 1a | Identification as a randomised trial in the title | 1 |
|  | 1b | Structured summary of trial design, methods, results, and conclusions (for specific guidance see CONSORT for abstracts) | 2-3 |
| Introduction | | | |
| Background and objectives | 2a | Scientific background and explanation of rationale | 5 |
|  | 2b | Specific objectives or hypotheses | 5 |
| Methods | | | |
| Trial design | 3a | Description of trial design (such as parallel, factorial) including allocation ratio | 6 |
|  | 3b | Important changes to methods after trial commencement (such as eligibility criteria), with reasons | n.a. |
| Participants | 4a | Eligibility criteria for participants | 6 |
|  | 4b | Settings and locations where the data were collected | 5, SAP |
| Interventions | 5 | The interventions for each group with sufficient details to allow replication, including how and when they were actually administered | 7, SAP and Supplement |
| Outcomes | 6a | Completely defined pre-specified primary and secondary outcome measures, including how and when they were assessed | 6-7 |
|  | 6b | Any changes to trial outcomes after the trial commenced, with reasons | n.a. |
| Sample size | 7a | How sample size was determined | 8-9 |
|  | 7b | When applicable, explanation of any interim analyses and stopping guidelines | n.a. |
| Randomisation: |  |  |  |
| Sequence generation | 8a | Method used to generate the random allocation sequence | n.a. |
|  | 8b | Type of randomisation; details of any restriction (such as blocking and block size) | n.a. |
| Allocation concealment mechanism | 9 | Mechanism used to implement the random allocation sequence (such as sequentially numbered containers), describing any steps taken to conceal the sequence until interventions were assigned | n.a. |
| Implementation | 10 | Who generated the random allocation sequence, who enrolled participants, and who assigned participants to interventions | n.a. |
| Blinding | 11a | If done, who was blinded after assignment to interventions (for example, participants, care providers, those assessing outcomes) and how | n.a. |
|  | 11b | If relevant, description of the similarity of interventions | n.a. |
| Statistical methods | 12a | Statistical methods used to compare groups for primary and secondary outcomes | 9-10 |
|  | 12b | Methods for additional analyses, such as subgroup analyses and adjusted analyses | 9-10 |
| Results | | | |
| Participant flow (a diagram is strongly recommended) | 13a | For each group, the numbers of participants who were randomly assigned, received intended treatment, and were analysed for the primary outcome | Fig.1 and Supplement |
|  | 13b | For each group, losses and exclusions after randomisation, together with reasons | Fig 1 |
| Recruitment | 14a | Dates defining the periods of recruitment and follow-up | recruitment |
|  | 14b | Why the trial ended or was stopped | n.a. |
| Baseline data | 15 | A table showing baseline demographic and clinical characteristics for each group | Tab 1 + Supplement |
| Numbers analysed | 16 | For each group, number of participants (denominator) included in each analysis and whether the analysis was by original assigned groups | All results |
| Outcomes and estimation | 17a | For each primary and secondary outcome, results for each group, and the estimated effect size and its precision (such as 95% confidence interval) | All results |
|  | 17b | For binary outcomes, presentation of both absolute and relative effect sizes is recommended | Done |
| Ancillary analyses | 18 | Results of any other analyses performed, including subgroup analyses and adjusted analyses, distinguishing pre-specified from exploratory | Done |
| Harms | 19 | All important harms or unintended effects in each group (for specific guidance see CONSORT for harms) | Done |
| Discussion | | | |
| Limitations | 20 | Trial limitations, addressing sources of potential bias, imprecision, and, if relevant, multiplicity of analyses | 16-17 |
| Generalisability | 21 | Generalisability (external validity, applicability) of the trial findings | 16-17 |
| Interpretation | 22 | Interpretation consistent with results, balancing benefits and harms, and considering other relevant evidence | discussion |
| Other information | | |  |
| Registration | 23 | Registration number and name of trial registry | 10-11 |
| Protocol | 24 | Where the full trial protocol can be accessed, if available | References |
| Funding | 25 | Sources of funding and other support (such as supply of drugs), role of funders | 10-11 |
